# Curcumin Alleviates Singapore Grouper Iridovirus-Induced Intestine Injury in Orange-Spotted Grouper (*Epinephelus coioides*)

**DOI:** 10.3390/antiox12081584

**Published:** 2023-08-09

**Authors:** Yue-Xuan Wang, Sui-Feng Xu, Ye-Wen Wang, Yun-Xiang Jiang, Qi-Wei Qin, Shi-Na Wei

**Affiliations:** 1 College of Marine Sciences, South China Agricultural University, Guangzhou 510642, China; wangyuexuan@stu.scau.edu.cn (Y.-X.W.); xusuifeng@stu.scau.edu.cn (S.-F.X.); wangyewen@stu.scau.edu.cn (Y.-W.W.); jiangyunxiang@stu.scau.edu.cn (Y.-X.J.); 2 Southern Marine Science and Engineering Guangdong Laboratory (Zhuhai), Zhuhai 528478, China; 3 Laboratory for Marine Biology and Biotechnology, Qingdao National Laboratory for Marine Science and Technology, Qingdao 266000, China

**Keywords:** Singapore grouper iridovirus, curcumin, intestine health

## Abstract

Singapore grouper iridovirus (SGIV) is a new ranavirus species in the Iridoviridae family, whose high lethality and rapid spread have resulted in enormous economic losses for the aquaculture industry. Curcumin, a polyphenolic compound, has been proven to possess multiple biological activities, including antibacterial, antioxidant, and antiviral properties. This study was conducted to determine whether curcumin protected orange-spotted grouper (*Epinephelus coioides*) from SGIV-induced intestinal damage by affecting the inflammatory response, cell apoptosis, oxidative stress, and intestinal microbiota. Random distribution of healthy orange-spotted groupers (8.0 ± 1.0 cm and 9.0 ± 1.0 g) into six experimental groups (each group with 90 groupers): Control, DMSO, curcumin, SGIV, DMSO + SGIV, and curcumin + SGIV. The fish administered gavage received DMSO dilution solution or 640 mg/L curcumin every day for 15 days and then were injected intraperitoneally with SGIV 24 h after the last gavage. When more than half of the groupers in the SGIV group perished, samples from each group were collected for intestinal health evaluation. Our results showed that curcumin significantly alleviated intestine damage and repaired intestinal barrier dysfunction, which was identified by decreased intestine permeability and serum diamine oxidase (DAO) activity and increased expressions of tight junction proteins during SGIV infection. Moreover, curcumin treatment suppressed intestinal cells apoptosis and inflammatory response caused by SGIV and protected intestinal cells from oxidative injury by enhancing the activity of antioxidant enzymes, which was related to the activation of nuclear factor erythroid 2-related factor 2 (Nrf2) signaling. Moreover, we found that curcumin treatment restored the disruption of the intestinal microbiota caused by SGIV infection. Our study provided a theoretical basis for the functional development of curcumin in aquaculture by highlighting the protective effect of curcumin against SGIV-induced intestinal injury.

## 1. Introduction

Due to the increasing demand for nutritious aquatic products, aquaculture has become an integral part of food security and economic growth. However, the invasion of viral pathogens has posed significant obstacles to the growth of aquaculture. Iridovirus is one of the most severe viral pathogens affecting marine and freshwater farmed fish, such as grouper (*Epinephelu* ssp.), largemouth bass (*Micropterus salmoides*), soft-shelled turtle (*Trionyx sinensis*), large yellow croakers (*Pseudosciaena crocea*), and so on [1,2,3]. In recent years, the novel ranavirus species Singapore grouper iridovirus (SGIV) has been isolated from diseased grouper [4,5]. It is extremely infectious to grouper farmed in China and Southeast Asia, and more than 90% of SGIV-infected fish died within a week, resulting in enormous economic losses for the aquaculture industry [6]. SGIV is a large icosahedral DNA-enveloped virus that primarily infects the epithelium and endothelial cells of the immune organs of grouper, such as the spleen, intestine, liver, and so on [6]. As one of the primary entry points for pathogens in aquatic-dwelling fish, the immune system of the intestine is an important line of defense against pathogens. Therefore, the changes in the intestine during SGIV invasion are of research interest.

The intestinal innate immune system is the first line of host defense against pathogens [7,8], so dysfunction of the intestinal barrier and injury to the mucosal immune system directly or indirectly contribute to pathogen invasion. A number of studies have indicated that virus invasion is associated with impairment of intestinal function. For example, the intestine is a major site of extrapulmonary infection in coronavirus disease 2019 (COVID-19), which is accompanied by intestine-blood barrier impairment, resulting in increased disease severity and mortality in humans (*Homo sapiens*) [9,10]. In rainbow trout (*Oncorhynchus mykiss*), infectious hematopoietic necrosis virus (IHNV) induces a comprehensive immune response of the intestine involved in toll-like receptor (TLR), RIG-I-like receptor (RLR), NOD-like receptor (NLR), nuclear factor kappa-B (NF-κB), Janus kinase (JAK)-signal transducer and activator of transcription (STAT), phosphoinositide 3-kinase (PI3K)-protein kinase B (Akt), interleukin 17 (IL-17), and advanced glycation end product (AGE)-advanced glycosylation end product-specific receptor (RAGE) signaling pathways [11]. Another piece of research indicated that the intestinal route is an important port of entry for tilapia lake virus (TiLV) in Nile tilapia (*Oreochromis niloticus*), and TiLV evades the host immune system by down-regulating the expression of the myxovirus-resistant (MX) gene in the intestine [12]. In addition, a report from Kyushu University found that in ginbuna crucian carp (*Carassius langsdorfii*), the intestine is an important site for generating virus-specific CD8^+^ cytotoxic T cells by vaccination with the inactivated vaccine of hematopoietic necrosis virus [13]. However, there are few studies on the association between SGIV infection and the intestine.

Curcumin, the main active ingredient of turmeric (*Curcuma longa*), possesses multiple biological activities such as antioxidant, antibacterial, and antiviral properties. Due to its chemical characteristics of low molecular weight and lipid solubility, it exhibits the ability to be absorbed through intestinal diffusion in a passive manner. An increasing number of reports have demonstrated the potential of curcumin to maintain intestinal health. For instance, curcumin improves insulin resistance, decreases blood sugar, and restores intestinal flora imbalances in diabetic rats (*Rattus norvegicus*) caused by a high-fat diet, as well as protecting the intestinal mucosal barrier [14]. Moreover, another study revealed that curcumin reduces the disruption of the intestinal epithelial barrier caused by deoxynivalenol by controlling the nuclear factor erythroid 2-related factor 2 (Nrf2)/tumor protein 53 (p53)-mediated apoptotic pathway and the NF-κB-mediated tight junction pathway in mice (*Mus musculus*) [15]. On the other hand, the phenolic hydroxyl group, which is part of the molecular structure of curcumin, is crucial to resisting damage by neutralizing free radicals. Several reports provided evidence that dietary curcumin improves intestinal permeability and inhibits oxidative stress in weaned piglets (*Sus scrofa*) [16]; curcumin ameliorates oxidative stress-induced intestinal barrier injury and modulates the AMP-activated protein kinase (AMPK) signal pathway to promote Parkin-dependent mitophagy for reducing mitochondrial damage in porcine intestinal epithelial cells [17]. In recent years, the value of curcumin in aquaculture has been demonstrated. Dietary curcumin addition can improve growth, antioxidant capacity, immune response, and intestinal histology in Nile tilapia and Rainbow trout [18,19,20]. Similar effects of curcumin on growth promotion, antioxidants, and anti-inflammatory properties have been demonstrated in climbing bass (*Anabas estudineus*) and crucian carp (*Carassius auratus*) [21,22]. Additionally, the addition of turmeric may improve the liver and gut health of golden pompano (*Trachinotus ovatus*) on a high-dietary-carbohydrate diet by attenuating oxidative stress and inflammation and decreasing lipid deposition [23]. However, it is unknown whether curcumin protects against intestinal injury caused by SGIV.

The present research was conducted to ascertain whether curcumin protected SGIV-induced intestinal damage by affecting the inflammatory response, cell apoptosis, oxidative stress, and intestinal microbiota in orange-spotted grouper. So far, this is the first study that highlights the protective effect of curcumin treatment against intestinal damage caused by marine fish DNA viruses, providing a theoretical foundation for the functional development of curcumin in aquaculture.

## 2. Materials and Methods

### 2.1. Ethics Statement

Our experiments with laboratory animals adhered to the South China Agricultural University’s (SCAU) institutional ethical guidelines. This study was approved by SCAU (ethical protocol code: 2020g009), and a certificate of approval is available upon request.

### 2.2. Virus and Animals

SGIV was isolated from sick fish, multiplied in grouper spleen (GS) cells, and then frozen at −80 °C [5].

Orange-spotted groupers (*Epinephelus coioides*) were procured from a fishery in Yangjiang City, Guangdong Province. Juveniles (8.0 ± 1.0 cm and 9.0 ± 1.0 g) were reared for acclimatization for two weeks in a seawater service system with 20 ± 2‰ salinity, 24 ± 1 °C water temperature. The dissolved oxygen was >5 mg/L, and the pH was 7.5 ± 0.2. The commercial feed was given to the fish once a day. The aforementioned rearing conditions remained unchanged during the trial. 

Prior to the experiment, groupers were selected at random for the detection of common fish viruses to ensure their health. RNA and DNA were extracted from the spleen, liver, and head kidney, respectively. RNA samples were reverse-transcribed to generate cDNA samples. The techniques of polymerase chain reaction (PCR) and agarose gel nucleic acid electrophoresis were used to detect SGIV in DNA samples, while red-spotted grouper nervous necrosis virus (RGNNV) and infectious spleen kidney necrosis virus (ISKNV) were detected in cDNA samples. According to the results, this batch of grouper was negative for SGIV, RGNNV, and ISKNV.

### 2.3. Experimental Design

Curcumin (MedChemexpress, Monmouth Junction, NJ, USA) was mixed with dimethyl sulfoxide (DMSO) (Solarbio, Beijing, China) to make 10 mM mother liquor, which was then diluted to 640 mg/L with sterile phosphate buffer saline (PBS). The same volume of DMSO as the curcumin mother liquor was added to PBS as a working fluid for gavage treatment.

A total of 540 groupers were randomly separated into 6 experimental groups, with 90 fish raised evenly in 3 tanks, as summarized in Table 1. Fish in groups administered gavage received 100 μL of the treatment at 4:00 p.m. daily for 15 days. Twenty-four hours after the last gavage, the fish in the infection groups were injected intraperitoneally with 100 μL of 10^7^ TCID_50_/mL SGIV dissolved in PBS. 

### 2.4. Sample Collection

When more than half of the groupers in the SGIV group died (the 6th day after SGIV injection), samples from each group were collected for follow-up experiments. Grouper infected with SGIV exhibited symptoms such as a darkened body color, an inability to swim, flotation due to a lack of oxygen, and near death. During the sampling procedure, all samples were obtained from live fish, and the clinical symptoms of the fish were not used to differentiate between samples. Blood samples were obtained by cardiac puncture from anesthetized fish (10 fish per tank, 30 fish in total per experimental group). Blood samples were collected and centrifuged, and the resulting serum was frozen at −80 °C. Before dissection, the fish brain was severed with anatomical shears to induce rapid death, and the intestines obtained were washed with cold PBS, flash-frozen in liquid nitrogen, and stored at −80 °C for future experiments. The intestinal contents of grouper were preserved at −80 °C before use. For paraffin embedding and sectioning in histopathology observation analysis and immunofluorescence analysis, the intestines of 9 fish (3 fish per tank, 9 fish in total per experimental group) were chosen at random. For the measurement of other indicators in this study, 9 fish (3 fish per tank, 9 fish in total per experimental group) were selected at random from each experimental group.

### 2.5. RNA Isolation and Real Time Quantitative PCR (qRT-PCR) Analysis

Using a tissue homogenizer to grind the intestine in pre-cooled PBS, the tissue homogenate was taken to extract total RNA with the Cell Total RNA Isolation Kit (FORE GENE, Chengdu, China). After that, the reverse transcription of RNA was implemented as described in the instructions of the ReverTra Ace qPCR RT Kit (TOYOBO, Osaka, Japan). For qRT-PCR analysis, an Applied Biosystems QuantStudio 5 Real-Time Detection System (ThermoFisher, Waltham, MA, USA) was utilized, and the conditions were as follows: 1 min at 95 °C, followed by 40 cycles for 15 s at 95 °C, 15 s at 60 °C, and 45 s at 72 °C. To ensure the specificity of detection at the conclusion of each cycle, a melting curve of the amplified product was created, and Table 2 shows the primers used in the study. Primers used in this study included housekeeping gene: β-actin; viral genes: Major coat protein (MCP), envelope 19 (VP19), lipopolysaccharide-induced TNF factor (LITAF); tight junction protein-related genes: Zonula occludens protein 1 (ZO-1), claudin-1, claudin-3, claudin-15, occludin; inflammatory factor genes: IL-1β, IL-6, IL-8, tumor necrosis factor-α (TNF-α), NF-κB; and antioxidant-related genes: Superoxide dismutase (SOD), catalase (CAT), glutathione peroxidase (GSH-PX), glutathione S-transferase (GST), Nrf2, Kelch-1ike ECH-associated protein l (Keap-1), heme oxygenase-1 (HO-1). The 2^−△△Ct^ method was used to compute the relative expression ratio of the selected gene against β-actin (reference gene). 

### 2.6. Histopathology Observations Analysis

The intestinal tissue was fixed in 4% paraformaldehyde, washed to remove excess fixative, dried in ethanol, and then paraffin-embedded. Hematoxylin and eosin were used to stain 5 μm-thick sections of the embedded materials. The photographs were captured using a microscope. The villus height, villus width, mucosal thickness, and layer thickness of grouper intestine were measured using Caseviewer 2.3.0 software (3DHISTECH Ltd., Budapest, Hungary). 

### 2.7. Nuclear/Cytosol Fractionation Assay

The intestine samples from different groups were subjected to nuclear protein and cytosolic protein fractionation using the commercial kit (BioVision, San Francisco, CA, USA) according to the protocols from the manufacturer. The separated proteins were used for Western blot assays.

### 2.8. Western Blot Analysis

The intestine tissue was ground with a tissue homogenizer in pre-chilled RIPA buffer (Beyotime Biotechnology, Shanghai, China), and the homogenized sample collected was centrifuged at 18,000× *g* for 15 min at 4 °C. After calculating the protein concentration with the bicinchoninic acid (BCA) method, the supernatant was incubated at 95 °C for 10 min with 5× loading buffer to denaturate the protein. The sodium dodecyl sulfate-polyacrylamide gel electrophoresis was used to isolate target proteins, which were then transferred to Immobilon polyvinylidene difluoride membranes. The membranes and skim milk were shaken for 2 h at room temperature, followed by an overnight incubation at 4 °C with the specific primary antibodies. After washing with PBST (phosphate-buffered saline–Tween), membranes were incubated for 30 min at room temperature with HRP-linked goat anti-rabbit IgG (1:5000 dilution, CST, Boston, MA, USA). Finally, immunoreactive bands were visualized using enhanced chemiluminescence (ECL) reagents. The specific primary antibodies used in the experiment are as follows: β-tubulin (1:2000 dilution, Abcam, Cambridge, UK), occludin (1:500 dilution, Bioss, Beijing, China), NF-κBp65 (1:500 dilution, Affinity BioSciences, Changzhou, China), cleaved caspase-3 (1:1000 dilution, CST, Boston, MA, USA), Nrf2 (1:500 dilution, Abcam, Cambridge, UK), Keap-1 (1:1000 dilution, CST, Boston, MA, USA), HO-1 (1:1000 dilution, CST, Boston, MA, USA) and NQO1 (1:200 dilution, CST, Boston, MA, USA), Lamin B1 (1:4000 dilution, Proteintech, Wuhan, China). Quantification was performed by ImageJ 1.51 software (NIH, Bethesda, MD, USA).

### 2.9. Determination of Intestinal Epithelial Cell Permeability

The experimental procedure was modified as previously described [24]. Before the experiment, sterile PBS was prepared with 0.8 mg/g of 4 kD FITC-dextran (Sigma-Aldrich, St. Louis, MO, USA). Six hours after the last feeding, we gavaged the groupers from each experimental group with 100 μL FITC-dextran in PBS, and 4 h later, their blood was collected and centrifuged to obtain the serum. The absorbance at 490 nm of the serum was measured using a microplate reader.

### 2.10. Measurement of Oxidative Stress Indices and Diamine Oxidase (DAO) Activity

In order to determine the oxidative stress index, tissue samples were homogenized in 0.01 M PBS, 10% homogenate was prepared, and sample protein was obtained by centrifuging the supernatant. The protein content of each sample was detected using BCA assay kits (Beyotime Biotechnology, Shanghai, China) that were commercially available. In the experiment to determine protein concentration, the samples were diluted to 2.5, 10, 50, and 100 times their initial concentration, and only results within the detection range of the standard curve were utilized to calculate the total protein content in the supernatant.

Reactive oxygen species (ROS), malondialdehyde (MDA), SOD, CAT, GSH-PX, and total antioxidant capacity (T-AOC) were measured in the intestine using commercial kits (Jiancheng Bioengineering Ltd., Nanjing, China) following the manufacturer’s instructions. The sample was diluted to 2, 5, 10, 20, 50, and 100 times the supernate for pre-testing, and the optimal dilution ratio was calculated according to the instructions. Reaction conditions related to the ROS detection assay were as follows: Samples were incubated with 20 μM 2,7-dichlorofluorescin diacetate (DCFH-DA) for approximately 1 h at 37 °C. Additionally, serum DAO activity was detected using the commercial kit from Jiancheng Bioengineering Ltd. (Nanjing, China). 

### 2.11. Immunofluorescence Analysis

Tissue samples were prepared in the same way as the histopathology observation assay described above. They were fixed in formaldehyde, embedded in paraffin, and sliced. Sectioned samples underwent antigen repair and were then incubated with serum. Afterwards, samples were incubated overnight with specific primary antibodies of ZO-1 (1:100 dilution, ABclonal, Wuhan, China), claudin-1 (1:200 dilution, Bioss, Beijing, China), occludin (1:200 dilution, Bioss, Beijing, China), cleaved caspase-3 (1:500 dilution, CST, Boston, MA, USA), Keap-1 (1:200 dilution, CST, Boston, MA, USA), HO-1 (1:500 dilution, CST, Boston, USA), and NAD (P)H quinone dehydrogenase 1 (NQO1) (1:200 dilution, CST, Boston, MA, USA) in the wet box. After being washed, samples were incubated with luciferin-conjugated goat anti-rabbit IgG (1:2000 dilution, CST, Boston, MA, USA) protected from light for 1 h. Fluorescent signals were visualized using fluorescence microscopy (Leica, Wetzlar, Germany), and quantification was performed by ImageJ 1.51 software (NIH, Bethesda, MD, USA).

### 2.12. Terminal-Deoxynucleoitidyl Transferase Mediated Nick End Labeling (TUNEL) Analysis

TUNEL assays were carried out to determine the DNA fragment of apoptosis intestinal cells using the DeadEnd Fluorometric TUNEL System Kit (Promega, Madison, WI, USA) following protocols from the manufacturer. Quantification was performed by ImageJ 1.51 software (NIH, Bethesda, MD, USA).

### 2.13. Caspases Activity Assay

Caspase activity assay kits (Beyotime Biotechnology, Shanghai, China) were used to detect the level of caspase activity in the intestine. In short, intestine tissues were ground in lysis buffer and then centrifuged. The contents of proteins in the supernatant were detected in BCA assays (Beyotime Biotechnology, Shanghai, China), and then the supernatant was incubated with the corresponding substrates. After 2 h, the absorbance was measured by a microplate reader.

### 2.14. Determination of Intestinal Microbiota

The intestinal microbiota of groupers was analyzed using 16S rRNA gene sequencing. Twelve hours after the last feeding, intestinal contents samples were collected. DNA extraction, sequencing, and data analysis were conducted according to the method described previously [25].

### 2.15. Statistical Analysis

The data were analyzed with GraphPad Prism 9 (GraphPad Software, Santiago, CA, USA) and displayed as the mean ± the standard error of the mean (SEM). SPSS version 20 (IBM, Armonk, NY, USA) was utilized to do a one-way analysis of variance. For statistical comparisons, Student’s *t*-test was utilized, and differences between means were deemed significant at *p* < 0.05.

## 3. Results

### 3.1. Curcumin Treatment Alleviated Intestine Damage Induced by SGIV

**Figure 1:**
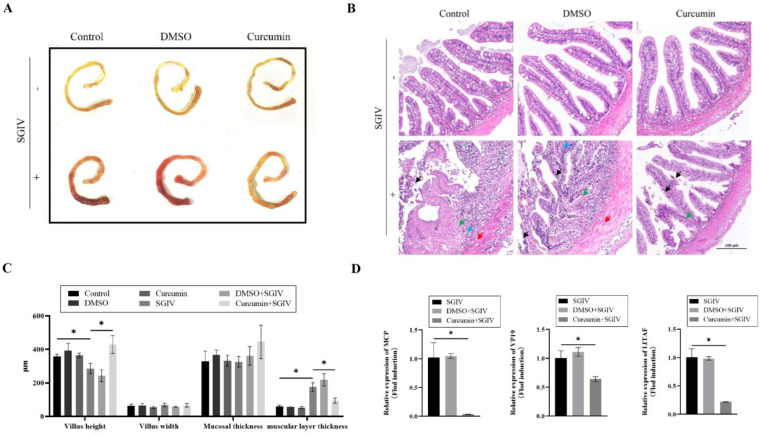
Curcumin treatment alleviated intestinal damage. (**A**) Intestine pictures. (**B**) H&E−stained liver pictures: Black arrow, epithelial exfoliation; red arrow, vacuolar degeneration of muscle fiber; green arrow, hypochromatic; blue arrow, inflammatory cell infiltration (scale bar = 100 μm). (**C**) Villus height, villus width, mucosal thickness, and muscular layer thickness of grouper intestine. The data are displayed as mean ± SEM (*n* = 9). (**D**) The expression of SGIV genes, including MCP, VP19, and LITAF, in intestine of SGIV-infected groupers. The data are displayed as mean ± SEM (*n* = 9). Student’s *t*-test: * *p* < 0.05.

Pathological changes in the intestine were examined to determine if curcumin treatment could aid in the repair of SGIV-induced damage. The intestine of groupers without SGIV was healthy and yellow, whereas the intestine of groupers infected with SGIV developed congestion and swelling (Figure 1A). In the H&E-stained sections of SGIV-infected grouper intestine, as indicated by the arrow, epithelial exfoliation, vacuolar degeneration of muscle fiber, cytoplasm puffing, and inflammatory cell infiltration were observed, while curcumin treatment ameliorated the aforementioned SGIV-induced changes (Figure 1B). Moreover, SGIV infection primarily affected the grouper intestine’s villus height and layer thickness but not its villus width or mucosal thickness (Figure 1C). In addition, the expression of SGIV genes was detected by qRT-PCR, showing that mRNA levels of SGIV-MCP, SGIV-VP19, and SGIV-LITAF in the grouper intestine of the curcumin treatment group were significantly lower than those in other groups (Figure 1D). These results demonstrated that curcumin treatment mitigated intestinal damage induced by SGIV.

### 3.2. Curcumin Treatment Prevented SGIV-Induced Intestinal Barrier Disruption

**Figure 2:**
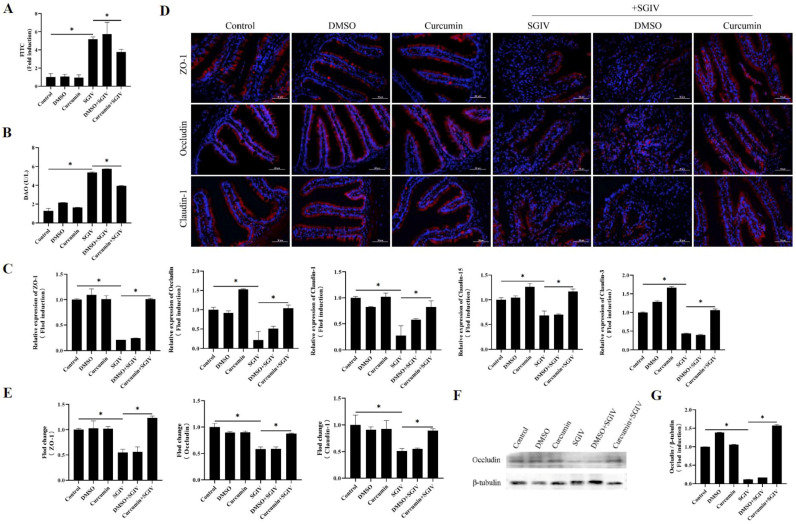
Curcumin treatment prevented SGIV-induced intestinal barrier disruption. (**A**) Permeability of intestinal epithelium was measured by FITC-Dextran gavage. The data are displayed as mean ± SEM (*n* = 9). (**B**) DAO activity in the serum. The data are displayed as mean ± SEM (*n* = 30). (**C**) The expressions of ZO-1, occludin, claudin-1, claudin-3, and claudin-15 were detected by qRT-PCR. The data are displayed as mean ± SEM (*n* = 9). (**D**) Representative images of immunohistochemistry staining with ZO-1, occludin, and claudin-1 antibodies in the intestine of groupers (scale bar = 50 μm). (**E**) Statistical analysis of the fluorescence intensity of ZO-1, occludin, and claudin-1. The data are displayed as mean ± SEM (*n* = 9). (**F**) The protein synthesis of occludin was detected by Western blot, and (**G**) the target/β-tubulin was calculated. The data are displayed as mean ± SEM (*n* = 9). Student’s *t*-test: * *p* < 0.05.

To inquire about the protective effects of curcumin treatment against SGIV-induced intestinal barrier disruption, the permeability of the intestinal epithelium and serum DAO activity were measured. We found that SGIV infection increased serum FITC-Dextran levels, which indirectly reflected epithelial permeability (Figure 2A), and simultaneously increased serum DAO activity (Figure 2B). Further experiments about the intestinal tight junction proteins related to the intestinal barrier indicated that, compared with the control group, the mRNA expression of ZO-1, occludin, claudin-1, claudin-3, and claudin-15 (Figure 2C) and the protein expression of ZO-1, claudin-1, and occludin (Figure 2D–G) decreased in the SGIV group, while curcumin treatment reversed these changes described above. The results suggested that curcumin treatment prevented intestinal barrier disruption caused by SGIV.

### 3.3. Curcumin Treatment Reduced Intestinal Cells Apoptosis

**Figure 3:**
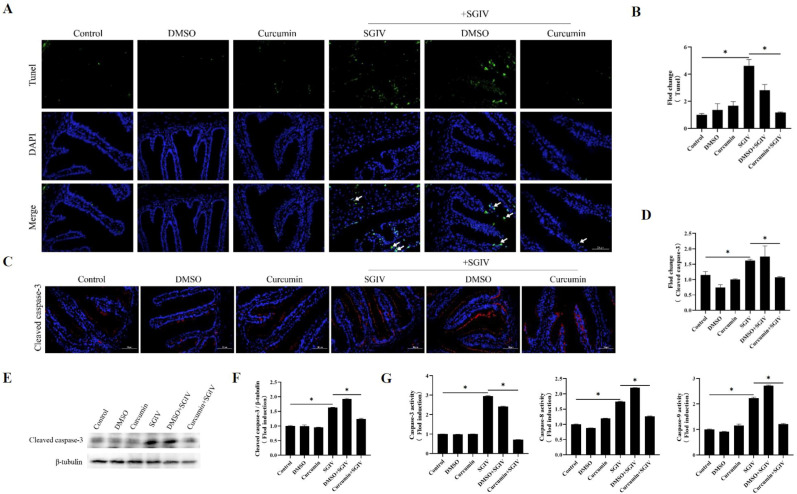
Effects of curcumin treatment on apoptosis in the intestine. (**A**) Detection of apoptosis by TUNEL assay. Green fluorescents indicated fractured DNA fragments, and blue fluorescents represented the nucleus. White arrows indicated apoptosis (scale bar = 50 μm). (**B**) Statistical analysis of the fluorescence intensity. The data are displayed as mean ± SEM (*n* = 9). (**C**) Representative images of immunohistochemistry staining with cleaved caspase-3 antibodies in the intestine of groupers (scale bar = 50 μm). (**D**) Statistical analysis of the fluorescence intensity of cleaved caspase-3. The data are displayed as mean ± SEM (*n* = 9). (**E**) The protein synthesis of cleaved caspase-3 was detected by Western blot, and (**F**) the target/β-tubulin was calculated. (**G**) The activity of caspase-3, caspase-8, and caspase-9. The data are displayed as mean ± SEM (*n* = 9). Student’s *t*-test: * *p* < 0.05.

TUNEL analysis confirmed intestine cell apoptosis, demonstrating that SGIV infection significantly caused DNA breakage in intestine cells and that curcumin treatment decreased DNA damage (Figure 3A,B). Moreover, SGIV infection enhanced the protein expression of cleaved caspase-3 (Figure 3C–F) as well as the activities of caspase-3/8/9 (Figure 3G), while curcumin treatment throttled these changes back. These results proved that curcumin treatment reduced intestinal cell apoptosis.

### 3.4. Curcumin Treatment Suppressed Inflammatory Response in Intestine

**Figure 4:**
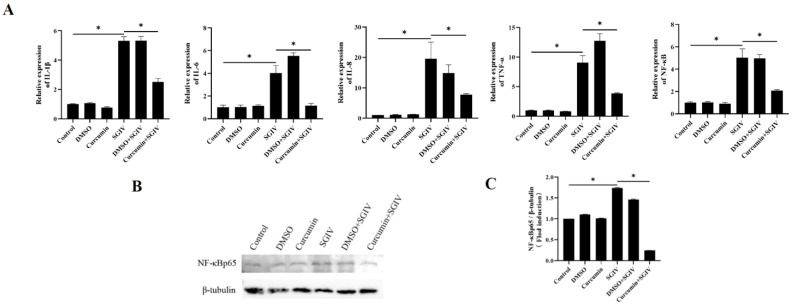
Effects of curcumin treatment on inflammatory reactions in the intestine. (**A**) The mRNA expressions of IL-1β, IL-6, IL-8, TNF-α, and NF-κB were detected. (**B**) The protein synthesis of NF-κBp65 was detected by Western blot, and (**C**) the target/β-tubulin was calculated. The data are displayed as mean ± SEM (*n* = 9). Student’s *t*-test: * *p* < 0.05.

To examine the effects of curcumin treatment on the inflammatory response of the intestine, the expressions of genes associated with the inflammatory response were detected using qRT-PCR and Western blot assays. The results implied that SGIV infection increased the mRNA expression of IL-1β, IL-6, IL-8, TNF-α, and NF-κB (Figure 4A) and the protein synthesis of NF-κBp65 (Figure 4B,C), while curcumin treatment slowed these changes down. The above findings suggested that curcumin treatment suppressed the inflammatory response triggered by SGIV in the intestine.

### 3.5. Curcumin Treatment Protected Intestinal Cells from Oxidative Injury

**Figure 5:**
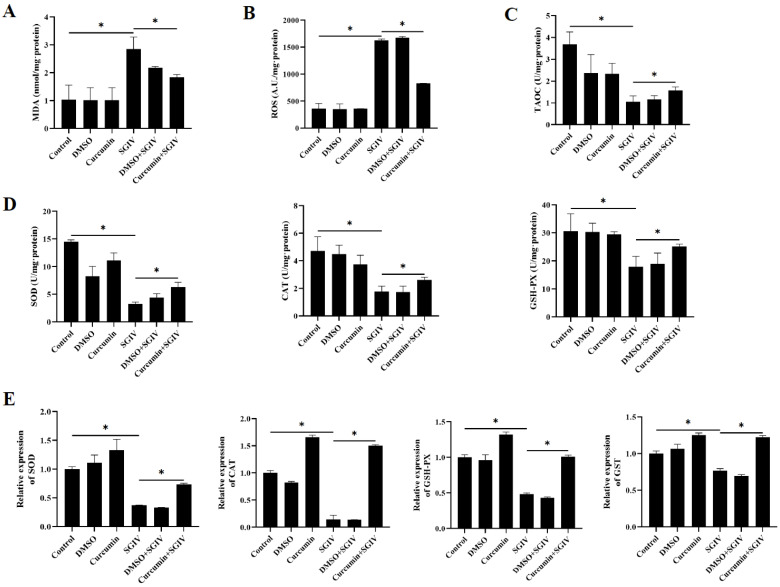
Effects of curcumin treatment on intestinal oxidative injury. (**A**) The contents of MDA in the intestine. (**B**) The contents of ROS in the intestine. (**C**) Total antioxidant capacity in the intestine. (**D**) Enzyme activities of SOD, CAT, and GSH-PX in the intestine. (**E**) The mRNA expression of SOD, CAT, GSH-PX, and GST. The data are displayed as mean ± SEM (*n* = 9). Student’s *t*-test: * *p* < 0.05.

The contents of MDA and ROS, as well as the levels of TAOC, SOD, CAT, and GSH-PX, were analyzed to determine the intestinal redox status. Our findings showed that in the SGIV group, the levels of MDA and ROS were higher than those in the control group (Figure 5A,B), and the activities of TAOC, SOD, CAT, and GSH-PX were lower than those in the control group (Figure 5C,D). In comparison to the SGIV group, curcumin treatment reversed these changes. Moreover, Figure 5E illustrated that the changes in mRNA expression of SOD, CAT, GSH-PX, and GST followed the same pattern as the changes in their enzyme activities. These findings suggested that curcumin treatment contributed positively to intestinal oxidation equilibrium.

### 3.6. Curcumin Treatment Promoted Nrf2 Signaling Activation via Down-Regulation of Keap-1

**Figure 6:**
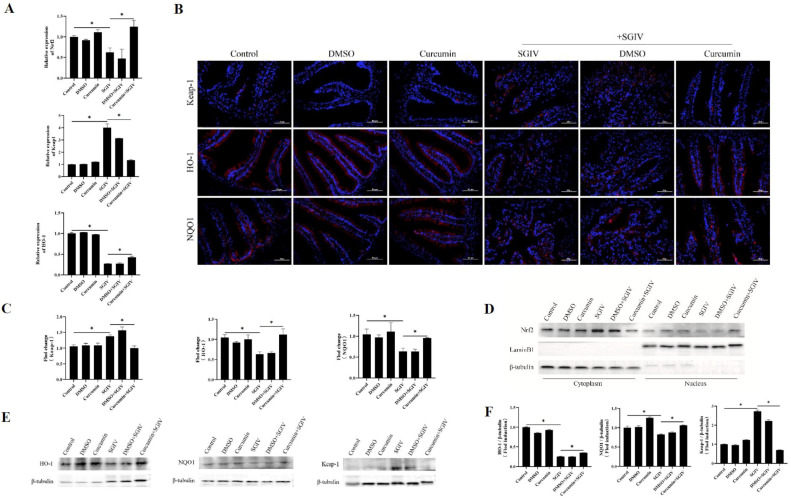
Curcumin treatment promoted Nrf2 signaling activation via turning Keap-1 down. (**A**) The mRNA expression of Nrf2, Keap-1, and HO-1. The data are displayed as mean ± SEM (*n* = 9). (**B**) Representative images of immunohistochemistry staining with Keap-1, HO-1, and NQO1 antibodies in the intestine of groupers (scale bar = 50 μm). (**C**) Statistical analysis of the fluorescence intensity. The data are displayed as mean ± SEM (*n* = 9). (**D**) The cytoplasmic and nuclear Nrf2 levels were detected by Western blot. β-tubulin and LaminB1 were the internal references for cytoplasmic and nuclear extracts, respectively. (**E**) The protein synthesis of HO-1, NQO1, and Keap-1 was detected by Western blot, and (**F**) the targets/β-tubulin was calculated. The data are displayed as mean ± SEM (*n* = 9). Student’s *t*-test: * *p* < 0.05.

To confirm whether the protective effects of curcumin treatment against oxidative injury in the intestine were related to Nrf2 signaling activation, the expression of Nrf2, Keap-1, HO-1, and NQO1 was measured. As shown in Figure 6, SGIV infection reduced the mRNA levels of Nrf2, HO-1 (Figure 6A), the fluorescence intensity of HO-1 and NQO1 in the intestine (Figure 6B,C), and the protein levels of HO-1 and NQO1 (Figure 6E,F), as well as Nrf2 protein expression in the nucleus (Figure 6D). In the meantime, SGIV infection increased the expression of Keap-1 at both the transcriptional and translational levels. Notably, compared with the SGIV group, curcumin treatment obviously increased nuclear Nrf2 levels and antioxidant enzyme levels, including HO-1 and NQO1, while inhibiting the expression of Keap-1. These findings implied that activation of the Nrf2 signaling pathway contributed to the protective effects of curcumin against SGIV-induced oxidative damage in the gut.

### 3.7. Effects of Curcumin Treatment on the Intestinal Microbiota in Grouper

**Figure 7:**
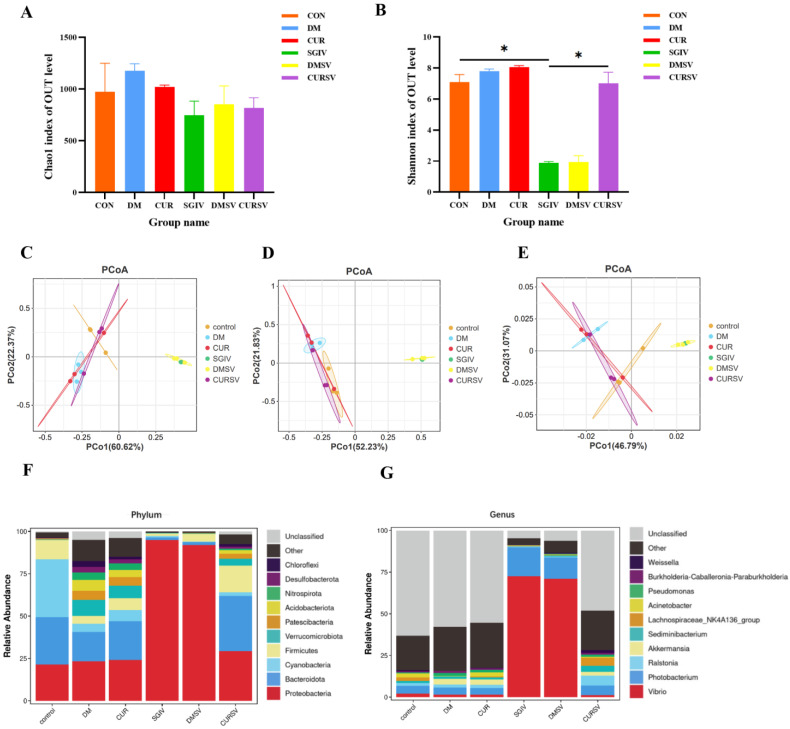
Effects of curcumin treatment on the intestinal microbiota in grouper. Differences in alpha-diversity of the intestinal microbiota, including (**A**) bacterial community richness (measured by the Chao1 index) and (**B**) bacterial community diversity (measured by the Shannon index). The data are displayed as mean ± SEM (*n* = 3). Student’s *t*-test: * *p* < 0.05. Principal coordinates analysis (PCoA) of the Bray−Curtis scores on the (**C**) phylum and (**D**) genus levels of the intestinal microbiota. (**E**) Principal coordinates analysis (PCoA) of the weighted UniFrac scores on the OUT level of the intestinal microbiota. Relative abundance at the (**F**) phylum and (**G**) genus levels of the intestinal microbiota. CON/control: control group; DM: DMSO group; CUR: curcumin group; SGIV: SGIV group; DMSV: DMSO + SGIV group; CURSV: curcumin+ SGIV group.

Differences in the microbial community were evaluated by the α-diversity, β-diversity, and species composition. Results of α-diversity evaluation showed that there was no significant difference in the Chao1 index among the six groups, whereas the Shannon index in the SGIV group decreased relative to the CON group (Figure 7A,B). Principal coordinate analysis (PCoA) was used to measure the β-diversity, revealing that, based on the PCoA analysis of the Bray–Curtis scores and the weighted UniFrac scores, samples clustered together according to treatment methods, with a clear separation between the control group and the SGIV group, while the composition and relative abundance of taxa were comparable between the control and CURSV groups (Figure 7C–E). The intestinal microbiota mainly belonged to Proteobacteria (21.4, 94.8, and 29.2%), Bacteroidetes (28, 1.6, and 32.6%), Cyanobacteria (34.2, 0.8, and 2.2%), Firmicutes (11.2, 1.5, and 15.8%), and Verrucomicrobia (0.4, 0.25, and 4.1%) in the CON, SGIV, and CURSV groups, respectively (Figure 7F). Further analysis at the genus level showed the percentages of *Vibrio* and *Photobacterium* in the SGIV group were 34.6 and 3.7 times higher than those in the CON group, respectively, and that curcumin treatment reversed these variations (Figure 7G). 

## 4. Discussion

The output of grouper aquaculture in China has surpassed 2 million tons, which has a high economic value, but disease outbreaks induced by viral pathogens present a significant obstacle to the mariculture industry. The intestinal tract serves as the primary site of pathogen invasion in fish. Pathogens may further propagate by interfering with the intestinal immune system and barrier function, ultimately gaining access to the bloodstream, which results in severe illness and mortality [26]. Teleost fish have a more decentralized gut-associated lymphatic system, where the mucosal immune system is extremely advantageous for fish living in a pathogen-rich aquatic environment [27]. Many fish species, such as the common carp (*Cyprinus carpio*), the European sea bass (*Dicentrarchus labrax*), and the Atlantic salmon (*Salmo salar*), have been shown to have immunocompetent cells in their intestinal mucosal immunity. This shows that the fish gut is an important part of preventing viruses from getting into the body [27,28,29]. Curcumin, the main active ingredient in turmeric, has been shown to be effective against fish viruses such as viral hemorrhagic septicemia virus and SGIV [30,31,32]. The accumulation of curcumin in intestinal mucosa and its pharmacokinetic interaction with drugs in intestinal cells suggest that intestinal cells may be the target cell type of curcumin action [33]. Consequently, we utilized the gavage method of curcumin to investigate its intestinal protective effect on SGIV-infected grouper. The assessment of intestinal health can be facilitated by utilizing intestinal morphology [34]. In the conducted study, it was observed that over 50% of SGIV-infected groupers exhibited notable swelling and congestion in the middle and posterior regions of the intestinal tract, while little such pathological phenomenon was observed in the intestinal tracts of the grouper in the curcumin + SGIV group. Subsequent pathological examination has demonstrated that SGIV infection led to a significant detachment of the epithelial cells, concomitant with the aggregation of inflammatory cells. The observed reduction in the height of the intestinal villi and the notable decrease in the thickness of the muscular layer were also indicative of severe damage to the intestinal tract. The intestinal epithelium is comprised of a monolayer of cells that functions as a discerning barrier against the entrance of exogenous noxious agents. Intramural domains of membrane-spanning proteins occludin and claudins are related to ZO-1, ZO-2, ZO-3, etc., and are associated with actin microfilaments, which jointly regulate the transportation function of intestinal barrier function [35]. Thus, the aforementioned tight junction proteins are crucial in upholding the integrity of the intestinal barrier. We discovered that infection with SGIV interfered with intestinal barrier function, as evidenced by elevated serum DAO levels and increased intestinal permeability, which might be attributable to the disruption of intestinal tight-binding protein expression and organization. Previously, oral supplementation with curcumin could lead to a twofold augmentation in intestinal alkaline phosphatase activity, resulting in a direct favorable impact on intestinal health [36]. Alternatively, the advantageous impacts of curcumin on the intestinal barrier have been demonstrated to enhance the function of the intestinal barrier through the conservation of ZO-1 and claudin-1 levels within the intestinal epithelium or mend the structure of the intestinal epithelium by decreasing the damage caused by lipopolysaccharides (LPS) to ZO-1, claudin-1, and claudin-7 [37]. Our findings indicated that the administration of curcumin resulted in a significant increase in villus height, preservation of tight muscle layers, maintenance of stable compact protein content, and enhancement of intestinal barrier function, thereby reducing the extent of intestinal damage caused by SGIV infection.

Intestinal inflammation is frequently observed in conjunction with impairment of the intestinal barrier. A reduction in intestinal alkaline phosphatase, which is a constituent of the luminal primary defense of the intestinal barrier, is observed in individuals suffering from inflammatory bowel disease [38]. Furthermore, sepsis mice exhibit indications of intestinal inflammation and impairment of the intestinal barrier [39]. Equally, in the process of fish farming, the presence of *Aeromonas hydrophila* or the pollutant trichlorfon causes impairment of the intestinal mucosal barrier function and subsequent inflammation of the intestines [40,41]. In the present study, it was observed that following SGIV infection, a significant accumulation of inflammatory cells occurred in the intestinal tissue of grouper, along with the up-regulation of the expression of pro-inflammatory cytokines including IL-1β, IL-6, IL-8, TNF-α, and NF-κB, indicating that SGIV invasion caused an intestinal inflammatory response. Studies related to rotavirus, reovirus, severe acute respiratory syndrome coronavirus 2 (SARS-CoV-2), astrovirus, and other viruses have extensively reported cases of intestinal inflammation caused by viral infection [42]. NF-κB is hypothesized to serve as a significant transcription factor implicated in inflammatory diseases of the intestinal tract. Upon activation, it is translocated to the nucleus, where it initiates transcription of target genes and triggers the generation of substantial amounts of pro-inflammatory cytokines. The continued aberrant synthesis of those cytokines within tissue may lead to inflammatory damage [43]. Hence, we speculated that SGIV infection might have disrupted the regular immune response in the intestinal cells via the activation of NF-κB signaling, thereby causing an upregulation of inflammatory markers. Interestingly, NF-κB is one of the targets of curcumin, and the primary mechanism of action for the anti-inflammatory effect of curcumin might be the inhibition of NF-κB [44]. According to reports, curcumin exhibits the attenuation of inflammation by modulating the NF-κB and JAK2/STAT3 signaling pathways in response to acute kidney injury [45]. Our findings indicated that curcumin treatment markedly inhibited the inflammatory reaction induced by SGIV and was concomitant with a reduction in the expression of NF-κB. Furthermore, we further found that SGIV infection induced intestinal cell apoptosis, as evidenced by the heightened presence of fragmented DNA and increased activity of caspase-3/8/9, providing supplementary support for the notion that SGIV caused harm to the intestine. Similarly, several viruses, including West Nile virus, porcine epidemic diarrhea virus, and mink enteritis virus, have been observed to trigger apoptosis in intestinal cells during infection [46,47,48]. However, it remains uncertain whether SGIV-induced apoptosis was a contributing factor to viral infection, as it can also function as a host-mediated mechanism to restrict infection, as seen in the case of reovirus in mice [49]. On the other hand, our study showed that curcumin treatment resulted in a decrease in DNA damage and the suppression of caspase-3/8/9 activation against SGIV-induced intestinal injury. Recently, emerging reports demonstrated that curcumin modulates multiple signal pathways in order to decrease cell apoptosis, which is regarded as an effective approach for its protection [50,51], and our discovery provided strong proof for this view.

Under normal physiological conditions, the level of ROS is controlled by endogenous antioxidants, which are involved in maintaining the balance of oxidative processes in the organism. However, oxidative stress induced by external stimuli can lead to the abnormal accumulation of ROS, causing impairment of intestinal barrier function and ultimately resulting in the development of intestinal diseases [52,53]. Numerous studies have demonstrated that ROS levels are implicated in and play a significant role in apoptosis, which possibly allowed us to analyze SGIV-induced apoptosis in intestinal cells [54,55]. So far, several viruses, including SARS-CoV, dengue virus, and rabies virus, have been known to induce oxidative stress during infection [56,57,58], analogous to our findings in this study that we discovered that after SGIV infection, MDA and ROS levels, which were positively correlated with oxidative damage in the intestine, increased, whereas T-AOC levels, which reflected the antioxidant capacity of tissues, decreased. In addition, the expression level and activities of enzymes (SOD, CAT, and GSH-PX) involved in the antioxidant defense system decreased after SGIV infection, whereas curcumin treatment was advantageous for the recovery of the intestinal antioxidant system, as indicated by its effect on the activity of a series of key antioxidant enzymes. Nrf2 is a crucial regulator of redox homeostasis in animal cells, and Keap-1 interacts with it in the cytoplasm to maintain its inactive state. Upon stimulation from the environment, Nrf2 dissociates from Keap-1 and enters the nucleus, where it bonds to antioxidant response elements and promotes the expression of downstream antioxidant enzymes, including NQO1 and HO-1 [59,60]. Previously, the viewpoint that Nrf2 exhibits antiviral properties was presented and substantiated in numerous instances in several studies. The research conducted by Bai et al. revealed that enterovirus 71 (EV71) infection elicits a heightened accumulation of ROS, impedes the activation of Nrf2, and facilitates the expression of Keap-1 at both the transcriptional and protein levels, indicating that EV71 specifically targets Nrf2 by instigating the production of its inhibitor [61]. An additional instance was demonstrated by Patra et al., in which they observed a decrease in Nrf2 via the proteasome in a fetal kidney cell line infected with rotavirus [62]. Although the precise mechanism of viral targeting of Nrf2 remains unknown, inhibiting Nrf2 still serves as a crucial viral strategy to facilitate efficient transmission. Simultaneously, the outcomes of our study served as an illustration to underscore this notion, where the expression of Keap-1 was elevated in the gut of SGIV-infected grouper, whereas the nucleoprotein level of Nrf2 and the total protein levels of HO-1 and NQO1 were considerably diminished. In addition, we found that curcumin treatment stabilized the expression of Nrf2 and its subsequent antioxidant subunits, which might be associated with the antioxidant properties of curcumin. Early studies suggested that the α,β-unsaturated carbonyl component of curcumin has the potential to attach to Keap-1 and enhance the stability of Nrf2 by obstructing ubiquitination and proteasomal degradation [63]. Overall, we proposed that the activation of Nrf2 was a crucial mechanism for the alleviation of SGIV-induced intestinal injury in grouper through curcumin treatment, but further investigation was required to determine the precise mode of action.

Following the process of replication, the virus proceeds to infiltrate the gastrointestinal tract, which functions as a conducive environment for the proliferation of microorganisms. The gut microbiome plays a crucial role in maintaining the host’s immune function, intestinal development, and homeostasis [64,65]. Drawing from previous research that showed curcumin tends to move around and build up in the intestine after being taken following oral administration [66], we thought that curcumin’s impact on the community of intestinal microbiota might constitute a potential mechanism underlying its protective effects on the gut. The present study revealed that SGIV infection resulted in a reduction in the diversity of the bacterial community. Subsequent findings demonstrated that SGIV had a significant impact on the abundance of several representative bacterial phyla, including Proteobacteria, Bacteroidetes, and Firmicutes. Notably, with the exception of Proteobacteria, which exhibited a 73.4% increase, the abundance of the other bacterial phyla decreased significantly after SGIV infection. Following SGIV infection, there was a notable increase of approximately 70% in the abundance of *Vibrio*, suggesting that the mortality of grouper during the latter stages of viral infection could potentially be attributed to mixed infection. Interestingly, many species of *Vibrio* are regarded as opportunistic pathogens for aquatic animals under stressful conditions, such as hypoimmunity [67]. According to reports, the infestation of white spot syndrome virus (WSSV) results in an escalation of *Vibrio* populations in the intestinal tract of Chinese mitten crabs (*Eriocheir sinensis*), and this can be attributed to the virus-induced interference with the host immune system, which in turn leads to the proliferation of opportunistic pathogens such as *Vibrio* [68]. An additional study potentially furnishes corroboration for the aforementioned hypothesis, indicating that the fatality rate of Pacific white shrimp (*Litopenaeus vannamei*) infected with WSSV and subsequently infected with *Vibrio* is in excess of 20% higher than that of shrimp solely infected with WSSV [69]. In addition, we found that, in comparison to the SGIV group, curcumin treatment reestablished the intestinal microbiota after its disruption, which was evidenced by an increase in the abundance of Bacteroidetes and Firmicutes as well as a decrease in the abundance of Proteobacteria. Likewise, it has been documented that the administration of curcumin demonstrates a noteworthy impact on the prevalence of Bacteroidetes, a beneficial bacterium, in mice [70]. Furthermore, several studies have demonstrated that curcumin effectively suppresses the development of colitis, possibly through the modulation of the microbiota [71,72]. Overall, these results suggest that curcumin treatment can protect the intestine by regulating the intestinal microbiota, which may contribute to preventing the invasion of viral pathogens. 

## 5. Conclusions

**Figure 8:**
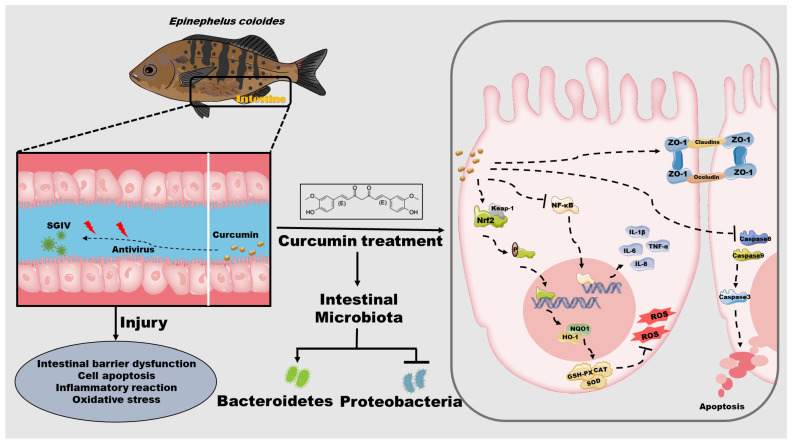
Schematic diagram representing the potential protection of curcumin on intestine in grouper by modulating intestinal barrier functions, cell apoptosis, inflammation, oxidative stress, and intestinal microbiota during SGIV infection.

The present study provided empirical support for the protective effects of curcumin against SGIV-induced intestinal injury in groupers (Figure 8). Through the promotion of tight junction proteins, which served to stabilize the intestinal barrier, as well as the reduction of cell apoptosis and suppression of the inflammatory response, curcumin significantly mitigated the SGIV-induced injury to the intestine. Curcumin also protected the intestine from oxidative stress by elevating the activities of antioxidant enzymes, which appeared to be associated with the activation of Nrf2 signaling. Notably, curcumin partially restored the intestinal flora disorder caused by SGIV infection, which may be the potential mechanism underlying its intestinal protective effect. Overall, these new findings provided valuable evidence that the use of curcumin is an effective method for reducing intestine injury and improving fish safety, suggesting its potential application in aquaculture.

## Figures and Tables

**Table 1:** Experimental design.

Groups	Days							
	1–15	16	17	18	19	20	21	22
Control								Sampled
DMSO	DMSO							Sampled
Curcumin	Curcumin							Sampled
SGIV		SGIV						Sampled
DMSO + SGIV	DMSO	SGIV						Sampled
Curcumin + SGIV	Curcumin	SGIV						Sampled

Control (no treatment); DMSO (gavage with DMSO); curcumin (gavage with curcumin); SGIV (injected with SGIV); DMSO + SGIV (gavage with DMSO and then injected with SGIV); curcumin + SGIV (gavage with curcumin and then injected with SGIV).

**Table 2:** Primers used in this study.

Primers	Sequences (5′–3′)	Gene Accession Number
β-actin-RT-F	TACGAGCTGCCTGACGGACA	AY510710.2
β-actin-RT-R	GGCTGTGATCTCCTTCTGCA	
MCP-RT-F	GCACGCTTCTCTCACCTTCA	YP_164167.1
MCP-RT-R	AACGGCAACGGGAGCACTA	
VP19-RT-F	TCCAAGGGAGAAACTGTAAG	YP_164114.1
VP19-RT-R	GGGGTAAGCGTGAAGACT	
LITAF-RT-F	GATGCTGCCGTGTGAACTG	YP_164231.1
LITAF-RT-R	GCACATCCTTGGTGGTGTTG	
EcZO-1-RT-F	GTCAAGTTCAAGAAGGGA	XM_033635847.1
EcZO-1-RT-R	TATTCAAAATGTGTGCGA	
EcClaudin-1-RT-F	AACAACCGCAGCAGAAG	XM_033650137.1
EcClaudin-1-RT-R	AGTGAATGGGTCGTAGAAG	
EcClaudin-3-RT-F	CCTTCATCCTGGCATCTCTGA	XM_033630592.1
EcClaudin-3-RT-R	GCACCTATCTCCCTCTTCTGT	
EcClaudin-15-RT-F	AAGTAGTGGCTCTGTTCCTGGGGTT	XM_033649884.1
EcClaudin-15-RT-R	GTTTTCATAGATGGTGGAGGTGGTG	
EcOccludin-RT-F	CCATATTTGCTTGTGTTGCCTC	XM_033622283.1
EcOccludin-RT-R	CATTGTAGTTCCCTCCGATTCC	
EcIL-1β-RT-F	AACCTCATCATCGCCACACA	EF582836.1
EcIL-1β-RT-R	AGTTGCCTCACAACCGAACAC	
EcIL-6-RT-F	GGTTGGTCCAAGGTGTGCTTA	KY012320.1
EcIL-6-RT-R	CTGGGATTGTCGAGGTCCTT	
EcIL-8-RT-F	GCCGTCAGTGAAGGGAGTCTAG	GU988706.1
EcIL-8-RT-R	ATCGCAGTGGGAGTTTGCA	
EcTNF-α-RT-F	GTGTCCTGCTGTTTGCTTGGTA	HQ011925.1
EcTNF-α-RT-R	CAGTGTCCGACTTGATTAGTGCTT	
EcNF-κB-RT-F	CTGCTGCCGAAGGTGGAGGGTGT	GU988726.1
EcNF-κB-RT-R	TGCGAACCTTACTACAGGCGACT	
EcSOD-RT-F	CAGCGGGACCGTGTATTTT	XM_033633905.1
EcSOD-RT-R	TTGTTGTGGGGGTTGAAGT	
EcCAT-RT-F	GGCAACAACACCCCCATT	XM_033635388.1
EcCAT-RT-R	CCAGAAGTCCCACACCAT	
EcGSH-PX-RT-F	CCCATCCCCTGTTTGTGTT	XM_033625833.1
EcGSH-PX-RT-R	CCTGGCTGAGGAGCTTCTT	
EcGST-RT-F	GGACCTGAATGGCTCACTGGAA	XM_033624859.1
EcGST-RT-R	GGGTCTCCCCTCAAACACATCC	
EcNrf2-RT-F	GTGGCAAGAACAAGGTAGC	XM_033617941.1
EcNrf2-RT-R	GTATTCGGAGGGGGAGTAG	
EcKeap-1-RT-F	TACGCTGTTTGGACTGCTCT	XM_033642809.1
EcKeap-1-RT-R	GCTGGACTCGGTGTTGTTTT	
EcHO-1-RT-F	CTACGACAGATTGGCAGAG	XM_033645634.1
EcHO-1-RT-R	GAAGGAGAAGAACGAAAGC	

## Data Availability

The data are contained within this article.
